# Elucidating Carbohydrate-Protein Interactions Using Nanoparticle-Based Approaches

**DOI:** 10.3389/fchem.2021.669969

**Published:** 2021-05-11

**Authors:** Dongyoon Kim, Nowras Rahhal, Christoph Rademacher

**Affiliations:** ^1^Department of Pharmaceutical Sciences, University of Vienna, Vienna, Austria; ^2^Max F. Perutz Laboratories, Department of Microbiology and Immunobiology, Vienna, Austria

**Keywords:** liposomes, quantum dots, gold nanoparticles, magnetic nanoparticles, endolysosomal sorting, ligand mobility

## Abstract

Carbohydrates are present on every living cell and coordinate important processes such as self/non-self discrimination. They are amongst the first molecular determinants to be encountered when cellular interactions are initiated. In particular, they resemble essential molecular fingerprints such as pathogen-, danger-, and self-associated molecular patterns guiding key decision-making in cellular immunology. Therefore, a deeper understanding of how cellular receptors of the immune system recognize incoming particles, based on their carbohydrate signature and how this information is translated into a biological response, will enable us to surgically manipulate them and holds promise for novel therapies. One approach to elucidate these early recognition events of carbohydrate interactions at cellular surfaces is the use of nanoparticles coated with defined carbohydrate structures. These particles are captured by carbohydrate receptors and initiate a cellular cytokine response. In the case of endocytic receptors, the capturing enables the engulfment of exogenous particles. Thereafter, the particles are sorted and degraded during their passage in the endolysosomal pathway. Overall, these processes are dependent on the nature of the endocytic carbohydrate receptors and consequently reflect upon the carbohydrate patterns on the exogenous particle surface. This interplay is still an under-studied subject. In this review, we summarize the application of nanoparticles as a promising tool to monitor complex carbohydrate-protein interactions in a cellular context and their application in areas of biomedicine.

## Introduction

**Figure 1:**
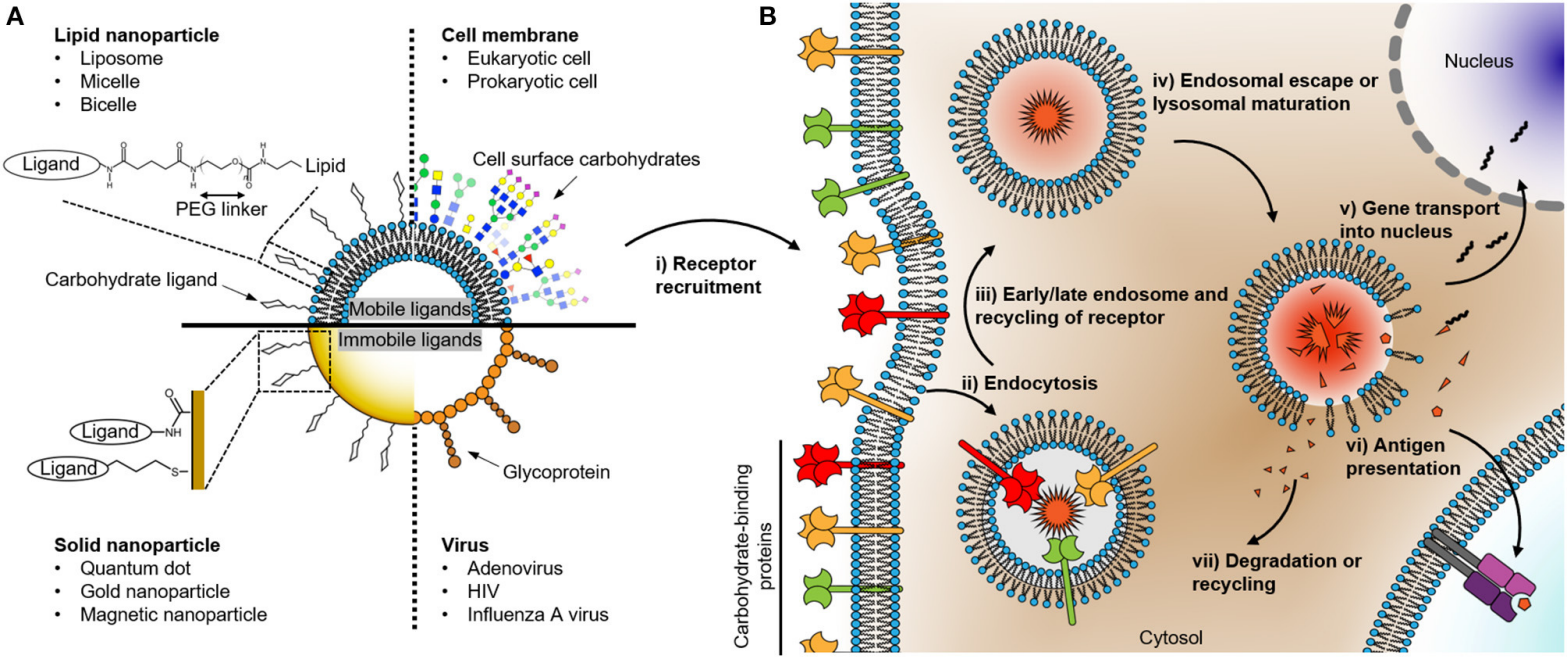
**(A)** Schematic drawing of nanoparticles coated with carbohydrates recognized by cell surface receptors. The right and left parts of the quadrants describe the surface structure of various organisms and their synthetic surrogates with representative conjugated ligand structures on the surfaces, respectively. The composition of the nanoparticle determines its chemo-physical properties which in turn affect the mobility, distribution, and localization of carbohydrate or glycoprotein ligand presented on the surface of the particle. **(B)** Uptake and degradation of carbohydrate organism in an antigen-presenting cell. The events following the recognition of the nanoparticle by the carbohydrate binding receptors consist of several steps: (i) receptor recruitment; (ii) endocytosis; (iii) endosomal maturation; (iv) the escape of cargo or degradation; the cargo can be either (v) transported into the nucleus in the case of a DNA or (vi) presented as an antigen on the cell surface. Finally, (vii) degradation or recycling of the remains.

All living cells, including viruses, are covered by a distinct pattern of carbohydrates on their surface. In the case of invading pathogens, this dense fur of carbohydrates is sensed by immune cells acting as sentinels equipped with a variety of carbohydrate-binding receptors. These receptors decode the carbohydrate signature of an individual incoming pathogen and the interplay between different signaling pathways elicited by the receptors orchestrates the cellular immune response. The response can result not only in immune cell maturation, cytokine secretion, but also particle uptake and processing leading to antigen presentation. This internalization process is initiated by the recognition of the carbohydrate-binding receptor and leads to the transportation and sorting of the particle in the endosomal pathway depending on the ligand types (Figure 1) (Cossart and Helenius, 2014; Mihov and Spiess, 2015; Fuchs et al., 2017; Jarvis et al., 2019). Therefore, understanding the relationship between the information encoded in carbohydrates present on the surface of an incoming microorganism and the elicited cellular response may lead to a better design of active pharmaceuticals for immunomodulation such as novel adjuvants or targeted delivery vehicles.

In the field of biomedical research, nanoparticles coated with carbohydrates have been used extensively over the past decade since they are excellent tools to study the pathophysiological roles of carbohydrates present on microorganisms. Their frequent use is likely due to their high versatility in many aspects: An individual nanoparticle can be coated with defined carbohydrates, which are recognized by a receptor on the immune cell, enabling the study of carbohydrate-specific responses and at the same time giving rise to valuable information on ligand multivalency in host-pathogen interactions. Additionally, nanoparticles are very powerful in comparison to other tools used to study carbohydrates on surfaces such as glycan microarrays, where two-dimensional surfaces are coated with defined carbohydrates (Rillahan and Paulson, 2011; Geissner et al., 2019). Nanoparticles can elicit a cellular response such as internalization and hence give a direct readout of biological activities. In particular, an individual nanoparticle is trackable either by fluorescence or light scattering imaging and provides a wealth of information on the underlying biology (Shen et al., 2017). Based on the type of nanoparticle, different aspects of biological readout can be improved. For example, nanoparticles that consist of lipids provide realistic carbohydrates-protein interactions mediated on cell-to-cell interfaces, which can therefore lead to more relevant readout in this respect. On the other hand, solid nanoparticles, composed of metal, semiconductors, or polymers, often possess and retain good biochemical stability and distinct, sensitive physical properties (e.g., optical or magnetic) in various physiological conditions and are therefore more suitable for quantitative analysis.

In this respect, it is useful to classify nanoparticles according to their biological composition. Consequently, in this review, we differentiate between solid and lipid nanoparticles (Figure 1A). Carbohydrate ligands on solid nanoparticles are immobile, while ligands on lipid nanoparticles can have a high level of mobility (Ramadurai et al., 2009). The diffusive properties of ligands on the surface of the particles can have a significant impact on the engagement and clustering of the carbohydrate receptor present on the cellular surface (Choi et al., 2019). Therefore, the mobility of ligands on nanoparticles can influence the free energy between ligands and receptors and thereby the endocytosis rate of the nanoparticle into cells (Schubertová et al., 2015; Zhdanov, 2017). In this perspective, it is interesting to compare the diffusion coefficient of glycolipids and glycoproteins present on viruses, bacteria, and eukaryotic cell surfaces. Glycoproteins on a virus can exhibit a sluggish diffusion (e.g., Env of HIV ~0.002 μm^2^/s, hemagglutinin of the influenza A virus ~0 μm^2^/s, the spike protein of adenovirus ~0 μm^2^/s; Benevento et al., 2014; Chojnacki et al., 2017; Vahey and Fletcher, 2019). To put this into perspective, the diffusion rates of glycoproteins on bacterial and eukaryotic cell surfaces can be as high as 0.020–1.000 μm^2^/s (Nenninger et al., 2014; Rodriguez-Rivera et al., 2017; Schavemaker et al., 2018). The low mobility of viral proteins is mainly due to the high lipid order of its membrane or the direct immobilization on the capsid such as for the adenoviral proteins (Chojnacki et al., 2017; Urbančič et al., 2018). Using nanoparticles as surrogates for incoming microorganisms suggests that solid nanoparticles can be ideal model systems for viruses. In contrast, relatively mobile ligands on lipid nanoparticles effectively emulate carbohydrate-protein interactions at the interfaces between eukaryotic cell surfaces or eukaryotic cell and bacterial surfaces. Therefore, it is important to choose an appropriate nanoparticle model system to understand physiologically relevant carbohydrate-protein interactions. In the following, we will focus on how solid and lipid nanoparticles have been exploited to study biomedical questions in glycobiology.

## Solid Nanoparticle Probes for Protein-Carbohydrate Interactions

### Characteristics of Solid Nanoparticles

Solid nanoparticles consist of polymeric, metallic, or semiconducting materials, which exhibit lower surface mobility compared with biological lipid membranes. Contrary to metallic and semiconducting nanoparticles, polymeric nanoparticles confer tunable mechanical modulus, which allows the emulation of various viral core stiffness which is correlated with viral maturation state and infectivity (Kol et al., 2007; Eshaghi et al., 2020). In addition, several pH-responsive self-assembled polymeric nanoparticles can be disassembled in low pH environments such as tumor region or endolysosome in the cell, providing targeted release of the cargo (Liao et al., 2018; Pan et al., 2018).

The bulky core space of solid nanoparticles can be occupied with fluorescence and/or magnetic materials thereby these particles have been used for example as localization probes for targeted delivery in *in vivo* research. Since, the reactive optoelectronic properties of nanoparticles can be moderated by the conjugation of organic molecules, such as polyethylene glycol (PEG), on the nanoparticle surface, these materials can be used in clinical applications, making them very attractive tools (Phillips et al., 2014; Bobo et al., 2016). These distinct properties of solid nanoparticles have been exploited in the field of basic glycobiological research and translated into biomedicine (Table 1).

**Table 1:** Classification of particles that have been used for probing carbohydrate-protein interactions.

**Particle type**	**Subtypes**	**Features**	**References**
Solid nanoparticle	Quantum dots (QDs)	Fluorescence resonance energy transfer reveals binding affinity between carbohydrates and C-type lectins	Guo Y. et al., 2017
		High quantum yield and long-term stability in acid condition allows monitoring QD-carbohydrate in cells	Chen et al., 2017; Tan, 2018; Fu et al., 2019
		Using QD-carbohydrate for targeted delivery	Kikkeri et al., 2009
	Gold nanoparticles (GNPs)	Label-free detection of carbohydrate-protein binding using optical scattering or surface plasmon resonance on GNPs	Reynolds et al., 2012; Tsutsumi et al., 2012; Richards et al., 2016; Sangabathuni et al., 2016
		Using carbohydrate-GNPs as a fluorescence quencher to investigate lectin binding	Budhadev et al., 2020
		Monitoring microscopic localization of GNPs in the cell through electron micrograph	Sangabathuni et al., 2016
		Using photodynamic effect of carbohydrate-coated GNPs for targeted cancer therapy	Tham et al., 2016; García Calavia et al., 2018
		Designing multifunctional gold nanoparticle for cancer therapy	Brinãs et al., 2012
	Magnetic nanoparticles (MNPs)	Exploiting transient magnetic birefringence to monitor carbohydrate-lectin interactions	Köber et al., 2014
		Detection of bacteria	El-Boubbou et al., 2007; Pera et al., 2010
		Using carbohydrate-coated MNPs and magnetic resonance imaging for targeted cell imaging	El-Boubbou et al., 2010; Farr et al., 2014
Lipid nanoparticle	Liposomes	Using carbohydrate-functionalized liposomes to investigate the effect of carbohydrate structure in carbohydrate-protein interactions	Freichel et al., 2019; Wamhoff et al., 2019
		Carbohydrate or glycomimetic ligand on liposome surface for targeted delivery	Kawasaki et al., 2013; Fehres et al., 2015; Ueki et al., 2015; Chen et al., 2016; Huber et al., 2016; Lai et al., 2018a; Su et al., 2018; Bartheldyová et al., 2019; Duan et al., 2019; Nycholat et al., 2019; Schulze et al., 2019; Wamhoff et al., 2019; Affandi et al., 2020
		Using glycoprotein-coated liposomes to mimic a pathogen or tumor and study immune cell activation	Hanson et al., 2015; Ingale et al., 2016; Broecker et al., 2018
		Leading cytokine release and/or cross-presentation of exogenous antigens	Kawasaki et al., 2013; Fehres et al., 2015; Huber et al., 2016; Affandi et al., 2020
		Conjugating dual carbohydrate ligand on liposome for increasing targeting efficacy	Lai et al., 2018b; Li et al., 2019

### Quantum Dots

Quantum dots (QDs) consist of semiconducting materials showing highly monodispersed particle distribution and variable size range (2–50 nm) depending on the synthesis method (McHugh et al., 2018). The high chemical stability and high quantum yield of QDs provide access to real-time monitoring of the endocytosis to endosomal sorting and escape, revealing discriminative carbohydrate sorting of cells. For example, QDs displaying carbohydrate-functionalized peptides are localized in the Golgi apparatus of normal lung tissue-derived cells after co-incubation. In contrast, functionalized QDs with non-glycosylated peptides are not localized in the Golgi apparatus. Furthermore, human lung cancer cells do not show the discriminative sorting whether the peptides contain the carbohydrate or not, but instead, all types of QDs are randomly distributed in the cytoplasm and the Golgi apparatus (Tan, 2018).

The processing of incoming exogenous particles decorated with carbohydrates is determined by the affinities between carbohydrates and their receptors. Initially, the interaction is weak for each individual, monovalent carbohydrate-protein recognition event. However, multivalent presentation of the carbohydrate ligand overcomes this threshold quickly. This affinity gain allows a Förster resonance energy transfer (FRET) from carbohydrate-coated QDs (donor) to a fluorophore-protein (acceptor) leading to a quantitative measure of recognition between the QDs and proteins (Guo Y. et al., 2017). The rate of energy transfer and thereby the acceptor fluorescence intensity is inversely proportional to the sixth of power of the distance, enabling quantification of bound_proteins on carbohydrates coated QDs. Overall, QDs have been served as a highly quantitative and robust tool for elucidating carbohydrate-protein interactions.

### Gold Nanoparticles

Gold nanoparticles (GNPs) have a variable size range (3–100 nm) and dispersity depending on the synthesis method (Hu et al., 2020). GNPs exhibit chemical inertness in physiological conditions and are readily functionalized through gold-thiol bonds as shown in Figure 1A (Alkilany and Murphy, 2010; Marradi et al., 2013; Toraskar et al., 2017). Moreover, GNPs have favorable optical properties. These particles are a very efficient light scatterer that changes their localized surface plasmon resonance frequency depending on the environment such as protein recognition of carbohydrate-coated GNPs (Tsutsumi et al., 2012; Richards et al., 2016). Therefore, GNPs have been utilized for label-free imaging and quantification of carbohydrate-protein interactions (Sangabathuni et al., 2016). In addition, GNPs have a strong fluorescent quenching property. This can be utilized since the emission light intensity from fluorescent dye-conjugated proteins is significantly reduced if the protein is bound on carbohydrates-coated GNPs (Budhadev et al., 2020).

Apart from their beneficial optical properties, individual GNPs can be visualized from transmission electron microscopy (TEM) and thereby elucidating nanoscale passaging and destination of carbohydrate GNPs inside cells (Le Guével et al., 2015). Moreover, a photodynamic effect of GNPs generates destructive reactive oxygen species that can suppress the targeted cells. In this respect, Russell and co-workers functionalized GNPs with lactose derivatives to target galectin-1, a carbohydrate binding protein overexpressed by malignant cells. They found a significant cell death in breast cancer cells upon co-incubation with functionalized GNPs and thereafter the irradiation of laser light. On the other hand, non-malignant human mammary epithelial cells were not affected by the same treatments (García Calavia et al., 2018). These distinct optical properties of GNPs in combination with carbohydrate coating have been showing potentials in biomedical applications.

### Magnetic Nanoparticles

Magnetic nanoparticles (MNPs), mostly composed of iron oxide, have variable size range (5–200 nm) and polydispersity index (~0.05–0.5) depending on the synthesis method (Guo X. et al., 2017; Gul et al., 2019; Panday et al., 2019; Wu et al., 2019). The attractive force between the magnetic nanoparticles and external magnetic fields allows a facile probe of carbohydrate and protein interactions. For example, Huang and Pieters group exploited carbohydrate-coated MNPs targeting adhesion proteins of pathogenic bacteria for water purification (El-Boubbou et al., 2007; Pera et al., 2010). Bacteria were bound to MNPs mediated by their bacterial adhesion proteins and the carbohydrates present on the MNPs. Furthermore, due to the multivalency effect, carbohydrate-coated MNPs and bacteria are further coagulated together. The bacteria-MNPs aggregates then can be harvested through an external magnetic field and further subjected to analysis. The increased hydrodynamic radius in turn affects the birefringence relaxation time in the external magnetic field, which can be readily measured by polarized light (Köber et al., 2014). Another interesting method utilizing MNPs is magnetic resonance imaging (MRI). Harms and co-workers were able to visualize the migration of MNPs bound to inflammatory cells in mice after intentionally initiating acute stroke. They used carbohydrate functionalized MNPs that target the proteins of inflammatory cells. Contrary to non-functionalized naked MNPs, which were accumulated in the ischemic vasculature, carbohydrate-coated MNPs were pronounced at the brain vasculature (Farr et al., 2014). This example shows nicely the potential application of MNPs in diagnostics.

## Lipid Nanoparticle Probes for Protein-Carbohydrate Interactions

### Characteristics of Lipid Nanoparticles

Lipid nanoparticles comprising liposomes, micelles, bicelles, and nanodiscs have been widely exploited and marketed as a vaccine platform (Li et al., 2015; Wang et al., 2018; Beltrán-Gracia et al., 2019). For example, targeting carbohydrate ligand-coated liposomes for cancer antigen delivery to antigen-presenting cell (APC) have shown promising results in cancer therapy (Lai et al., 2018a; Affandi et al., 2020). Most recently, lipid nanoparticles got an emergency use authorization as an mRNA carrier for vaccines against the severe acute respiratory syndrome coronavirus 2 (SARS-CoV-2) (Forni and Mantovani, 2021).

Compared to solid nanoparticles, the surface of lipid nanoparticles is more mobile due to the fluidic properties of the lipids. Depending on the composition and environment of the lipid bilayer (e.g., giant unilamellar vesicle and supportive/free-standing lipid bilayer), the diffusion constant of lipid varies from 0.1 to 8.0 μm^2^/s (Machán and Hof, 2010). The mobile lipids confer Brownian movement of carbohydrate ligands on the lipid surface and thereby enable multivalent bindings in a confined domain, which is crucial for downstream signaling and uptake of immune cells (Goodridge et al., 2011). In this respect, together with the favorable biocompatibility, lipid nanoparticles have been widely used, not only as model systems in glycobiology, but also to assist targeted delivery platforms for active delivery processes (Zahednezhad et al., 2019). Although many types of lipid nanoparticles have been conjugated with carbohydrates for the sake of targeted delivery, we will exclusively review on liposome since its generality.

### Liposomes

Liposomes are spherical phospholipid nanoparticles being around 100–200 nm in size and with a polydispersity index value of 0.2 (Soema et al., 2015). Note that the size and polydispersity can vary widely depending on the composition and formulation method. Liposomes can easily be decorated with carbohydrates by formulating defined structures present on lipids. In particular, not only the types of ligands but also their densities on the liposomes can be adjusted by altering the ratio between the ligand-conjugated and ligand-free lipids during the formulation process. Additionally, the bottom-up self-assembly formulation methods of liposomes enable tunable and independent control over their size, composition, and zeta potential. Therefore, liposomes have been used as well-defined probes for carbohydrate-protein interactions in many aspects. In this regard, Song and co-workers prepared a dual carbohydrate ligand-conjugated liposome to target Kupffer cells, macrophages localized in the lung. They found that the correctly adjusted ratio between fucose and mannose moieties present on the liposome provides the highest uptake in Kupffer cells (Lai et al., 2018b). Furthermore, in our laboratory, we explored liposomes as carriers for glycomimetic ligands that provide high specificity for defined carbohydrate receptors. A targeting ligand for human Langerin was developed that showed highly specific delivery of liposomal formulations to Langerhans cells, an antigen-presenting cell subset present in human skin (Wamhoff et al., 2019; Bellmann et al., 2021). Thereafter, the Langerin targeting liposomes were equipped with pH-sensitive fluorescent dyes via the co-formulation with the pH-sensitive fluorescent dye-lipid conjugates. The multifunctional liposomes in combination with live cell imaging allowed real-time monitoring of dynamics and endolysosomal maturation of endocytosed liposomes in Langerin expressing cells (Schulze et al., 2019). In parallel, Hartmann and co-workers applied functionalized liposomes with a lactose-based carbohydrate ligand, varying the linker length and valences. In a binding assay to galectin-3, the number of lactose molecules and its spacing to liposomes were the dominant factors affecting the recognition process. Furthermore, lactose functionalized liposomes exhibit an increased affinity to galectin-3 compared with dispersed lactose ligands, indicating the role of multivalency in carbohydrate-protein interactions (Freichel et al., 2019).

Lipid nanoparticles, unlike solid nanoparticles, can be degraded and recycled back onto the cell membrane. Therefore, liposomes have been used widely as a delivery platform into the cell (Bulbake et al., 2017). Various carbohydrate receptors have been exploited for such approaches, amongst them are prominently the Siglecs and C-type lectins. By targeting receptors present on antigen-presenting cells, a wide range of immunological responses have been studied *in vitro* as well as *in vivo*, such as cross-presentation of cargo antigen with hormonal response leading to subsequent T cell activation (Kawasaki et al., 2013; Fehres et al., 2015; Huber et al., 2016; Lai et al., 2018a; Affandi et al., 2020). To elicit immune cell activation, the carbohydrate- or glycomimetic-coated liposomes must undergo endocytosis and endosomal sorting (Figure 1B). Each of these processing steps is likely sensitive to the nature of the ligand/receptor pair. For example, liposomes modified with Lewis-Y, a difucosylated oligosaccharide glycoconjugate found at the cell surface, bound to both langerin and DC-SIGN positive cells. However, endocytosis and cross-presentation of the formulated cargo antigen were only successfully shown in DC-SIGN positive cells. On the other hand, liposomes coated with modified Lewis-Y with synthetic peptide exhibit increased endocytosis and cross-presentation in both langerin and DC-SIGN positive cells (Fehres et al., 2015). Overall, liposomes have been used in various aspects to investigate carbohydrate-protein interactions and translating these into biomedical applications such as immunomodulation.

## Future Directions and Summary

Being far from comprehensive, we hope that we could give the impression that the utilization of nanoparticles in fundamental glycobiological research has already given rise to many applications in biomedicine. The distinct features of lipid and solid nanoparticles make them effective tools to investigate cell-to-cell and host-pathogen interactions. However, in spite of the obvious difference in ligand mobility on lipid vs. solid nanoparticle surfaces, it is still not fully understood how such mobility of the carbohydrate ligand influences its initial binding and consequently the endolysosomal sorting in the cells. This is of particular interest since it is plausible that spontaneous thermal fluctuation of mobile carbohydrate ligands on nanoparticle surfaces subsequently accelerates the number of valences between the ligands and their receptors after the initial binding on the cell, and thereby influence the endocytosis rate of the nanoparticles (Gao et al., 2005). Therefore, further quantitative research is required to understand the role of ligand mobility in carbohydrate-protein interactions.

The main challenge facing solid nanoparticles is toxicity arising from the reactive metallic surface which generates reactive oxygen species in the cell (Attarilar et al., 2020). Although PEGylation of nanoparticles provides a longer circulation time in blood and minimizes the toxicity as of being a protective layer, it also brings disadvantages such as reduction in transfection potency and cellular uptake, and also immunogenic reactions (Garay and Labaune, 2011). For this reason, many studies have been used PEG alternatives such as natural polysaccharides or a synthetic polymer-based protective coating (Ahmed and Aljaeid, 2016; Libralato et al., 2017; Laksee et al., 2018; Fam et al., 2020). But the immunogenic problem has not been addressed yet.

In summary, we have summarized recent progress of the application of carbohydrate-coated nanoparticles probing carbohydrate-protein interactions. Solid nanoparticles comprising QDs, GNPs, and MNP have been used mainly for quantitative carbohydrate-protein affinity measurements and subcellular trackers. Lipid nanoparticles have been extensively exploited as cellular modulators for instance via targeted delivery. Altogether, carbohydrate-coated nanoparticles allow us to investigate how cells process complex carbohydrate information from carbohydrate-binding proteins, and therefore could lead to the development of novel therapeutics such as adjuvants or vaccines.

## Author Contributions

DK and CR conceptualized the approach. All authors wrote the article.

## Conflict of Interest

CR is shareholder of Cutanos GmbH, Vienna. The remaining authors declare that the research was conducted in the absence of any commercial or financial relationships that could be construed as a potential conflict of interest.
